# Apigenin attenuates myocardial ischemia-reperfusion injury through miR-448/SIRT1 axis

**DOI:** 10.22038/ijbms.2025.80172.17365

**Published:** 2025

**Authors:** Chenchen Tian, Bo Yu, Yibo Liu, Zhipeng Diao, Yue Wang, Jianmei Zhou

**Affiliations:** 1 School of Integrative Medicine, Tianjin University of Traditional Chinese Medicine, Tianjin 301617, China; 2 Department of Cardiology, Tianjin Union Medical Center, Nankai University Affiliated Hospital, Tianjin 300121, China; 3 Department of Cadre Health Care, Zhejiang Hospital, Hangzhou, Zhejiang 310013, P.R. China

**Keywords:** Apigenin, Apoptosis, Autophagy, MiR-448, OGD/R

## Abstract

**Objective(s):**

Myocardial ischemia/reperfusion injury (MIRI) is the primary pathological injury following ischemic cardiomyocyte therapy, but there are few effective treatments available for MIRI. Apigenin (API) is an active ingredient of herbal medicine. Our study aims to verify whether API regulates autophagy and apoptosis against MIRI via miR-448/Sirtuin-1 (SIRT1) axis.

**Materials and Methods:**

MTT, SOD, and LDH assays were used to measure cell viability, oxidative stress injury, and cell damage, respectively. RT-qPCR, western blot, and ELISA were used to measure RNA and protein expression levels.

**Results:**

Compared with the control group, cell viability and SOD levels in the cells of the OGD/R group were significantly decreased, LDH release in the cells was significantly increased, the level of miR-448 in the cells was significantly increased, the levels of SIRT1 mRNA and protein in the cells were significantly increased, the expression of LCII/I and Bcl-2 proteins in the cells were significantly down-regulated, and the expression of p62, Bax proteins in the cells and caspase-3 protein in the cell supernatant were significantly up-regulated. Compared with the OGD/R group, the above indicators were significantly reversed in the OGD/R+API group and the OGD/R+miR-448 inhibitor group. Compared to the OGD/R+miR-448 inhibitor group, the above indicators were significantly reversed in the OGD/R+miR-448 inhibitor+EX527 (SIRT1 inhibitor) group. Compared to the OGD/R+API group, the above indicators were significantly reversed in the OGD/R+API+miR-448 mimic group, OGD/R+API+EX527 group, and OGD/R+API+CA-5f (autophagy inhibitor) group.

**Conclusion:**

API regulates autophagy and apoptosis via the miR-448/SIRT1 axis against MIRI.

## Introduction

Ischemia/reperfusion injury (IRI) is defined as a further exacerbation of ischemic injury when blood flow is restored after a brief interruption (1). When patients with acute myocardial infarction receive reperfusion therapy, restoring blood flow to the ischemia myocardium induces myocardial ischemia/reperfusion injury (MIRI), leading to adverse cardiac remodeling, reperfusion arrhythmias, and myocardial stunning (2). However, current treatments against MIRI, such as ischemic pretreatment and post-ischemic adaptation, still have certain limitations in protecting the elderly myocardium (3, 4). Therefore, discovering an effective drug or method against MIRI is urgently needed.

Apigenin (API) is a flavonoid compound. It is mainly derived from the umbrella family of parsley and is also found in other plants such as chamomile, celery, perilla, and verbena (5, 6). It has a variety of biological activities, including anti-inflammatory and cardiovascular protective effects (7). In 2017, a relative study found that API protected against renal IRI via the activation of the Janus kinase 2 (JAK2)/signal transducer and activator of the transcription 3 (STAT3) signaling pathway (8). In 2021, He *et al*. discovered that API attenuated renal IRI inflammation via the miR-140-5p/chemokine12 (CXCL12)/nuclear factor κB (NF-κB) signaling pathway (9). Moreover, several studies reported that API attenuated MIRI by regulating Notch1/Hes1 signaling and miR-15b (10, 11). MicroRNAs (miRNAs) are classical non-coding RNAs that bind to the 3’UTR of target mRNAs to induce mRNA degradation (12). miRNA-448 expression significantly increased in myocardial infarction rats, and miRNA-448/vascular endothelial growth factor A (VEGFA) suppressed the fatty acid synthase (FAS)/FAS-L signaling pathway against cardiomyocyte hypoxia injury (13). Furthermore, Nie* et al. *found that LncRNA AK006774 expression decreased in the MIRI model, and the LncRNA AK006774/miR-448/B-cell lymphoma 2 (Bcl-2) signaling pathway played an essential role in the development of MIRI (14). The previous research indicates that miRNA-448 plays a crucial role in MIRI.

Autophagy is crucial for biological homeostasis, providing valuable energy during times of nutrient deprivation and removing reactive oxygen species (ROS), damaged proteins, and dysfunctional organelles to maintain homeostasis, so the decline in autophagy efficiency is usually associated with the development of many diseases, such as cardiomyopathy, infection, and cancer (15). However, excessive autophagy leads to cell death. Since cardiomyocytes are poor regenerators, excessive autophagy is dangerous for cardiomyocytes. (16). Moderate endoplasmic reticulum stress can promote autophagy and restore the system to the previous or new homeostasis, whereas excessive stress can lead to apoptosis (17). Recently, a study demonstrated that Suxiao Jiuxin Pill inhibited autophagy and apoptosis from attenuating MIRI injury via regulation of the AlkB homolog 5 (ALKBH5)/glycogen synthase kinase 3β (GSK3β)/mammalian target of rapamycin (mTOR) signaling pathway (18). Mitophagy plays a key role in mitochondrial quality control in cardiomyocytes, and Zheng *et al*. found that resveratrol enhanced mitophagy to improve cardiac mitochondrial quality against MIRI (19). The above studies suggest that autophagy in MIRI has a complex mechanism. Sirtuin-1 (SIRT1) is a highly conserved nicotinamide adenine dinucleotide (NAD^+^)-dependent histone deacetylase involved in cellular autophagy. Activation of SIRT1 can attenuate pathological damage to MIRI. Growing studies have shown that SIRT1-mediated autophagy plays an important role in MIRI by modulating various autophagy-related pathways, such as microtubule-associated protein light chain 3 (LC3), autophagy-related proteins (ATGs) and forkhead box proteins (FOXOs) (20). A study found that resveratrol suppressed apoptosis and promoted autophagy against MIRI via the SIRT1 signaling pathway (21). In this study, we hypothesize that API regulates autophagy and apoptosis against MIRI via the miR-448/SIRT1 signaling pathway. In basic medical experiments, oxygen and glucose deprivation, reoxygenation, and glucose (OGD/R) damage models in H9c2 cells are often used to mimic MIRI *in vivo* (22). Herein, the OGD/R model was established using H9c2 cells to explore the molecular mechanisms of API against MIRI *in vitro*, laying the foundation for the clinical application of API in the treatment of MIRI.

## Materials and Methods

### Reagents and cell line

Cobalt chloride (CoCl_2_) (Aladdin Biochemical Technology Co., Ltd, Shanghai, China), DMEM high-sugar medium and DMEM sugar-free medium (Gibco Company, USA), FBS (South American, LONSERA company), API (Yuanye Biotechnology Co., Ltd, Shanghai, China), 3-(4, 5-dimethyl-2-thiazolyl)-2, 5-diphenyl-2-H-tetrazolium bromide (MTT) powder (Solaibao Biotechnology Co., Ltd, Beijing, China), caspase-3 enzyme-linked immunosorbent assay (ELISA) kit (ET Life Sciences R&D Co., Ltd), superoxide dismutase (SOD) and lactate dehydrogenase (LDH) measure kit (Jiancheng Bioengineering Research Institute, Nanjing, China), Trizol reagent, ABScript II cDNA Strand Synthesis Kit, Universal SYBR Green Fast qPCR Mix (Aibotech Biotechnology Co., Ltd, Wuhan, China), SIRT1 primers (Dingguo Changsheng Biotechnology Co., Ltd, Beijing, China), miRNA reverse transcription and qPCR kits, miR-448 primers (Hefei, Zhien Biotechnology Co., Ltd), BCA protein quantitation assay kit (Biyuntian Biotechnology Co., Ltd), Primary antibodies: hypoxia inducible factor 1α (HIF-1α) (USA, Affinity Bioscience), SIRT1, Sequestosome 1 (SQSTM1/p62), LC3, Bcl-2, Bcl-2-associated X protein (Bax), caspase-3, β-actin (Aibotech Biotechnology Co., Ltd, Wuhan, China), Secondary antibodies: horseradish peroxidase (HRP) linked anti-rabbit IgG and anti-mouse IgG (Soleibao Biotechnology Company, Beijing, China), Lipofectamin^TM^2000 (Invitrogen Company, USA), OPTI MEM medium (Gibco Company, USA), miR-448 mimic/inhibitor or NC (Jima Pharmaceutical Technology Co., Ltd, Shanghai, China), SIRT1 inhibitor EX-527 and autophagy inhibitor CA-5f (MedChemExpress Company, China). Rat cardiomyocyte H9c2 cell line obtained from the Cell Bank of the Chinese Academy of Sciences and incubated with DMEM high glucose medium supplemented with 10% FBS, 100 IU/ml penicillin, and 100 μg/ml streptomycin.

### Molecular docking

From the PubChem database (https://pubchem.ncbi.nlm.nih.gov/) the “2D Structure, SDF” style of the API was downloaded. The SDF style was converted to the PDB style using the Open Babel GUI software. The SDF style of the target proteins was downloaded from the UniProt database (https://www.uniprot.org/) and the PDB database (https://www.rcsb.org/). The PDB ID numbers were: LC3A(4ZDV), LC3B(5D94), SIRT1(4ZZI), Bax(6TRR), Bcl-2(2W3L), p62(6MJ7), caspase-3(1QX3). Solvent, organic, and protein residues were removed using Pymol.exe software. The API was docked to the target proteins in AutoDock Tools 1.5.6 software. The binding affinity between the API and the target proteins was evaluated using the minimum binding energy. Finally, the minimum binding energy file was visualized in the Pymol software. 

### Determination of simulated low oxygen concentration of CoCl2 in H9c2 cells

The experiment was divided into three groups: The control group, the 1% O_2_ group, and the 0.1, 0.2, 0.3, 0.4, 0.5, 0.6, 0.8, 1.0, and 1.2 mM CoCl_2 _group. H9c2 cells in the logarithmic growth phase were seeded in six-well plates at 2×10^5^ per well, and the medium was discarded after 24 hr of incubation at 37 °C, 5% CO_2_. It was replaced with 1% FBS medium to synchronize the cells for 24 hr. These cells were then subjected to hypoxia for 24 hr in a 37 °C three-gas incubator (94% N_2_, 5% CO_2_, 1% O_2_) or treatment with 0.1, 0.2, 0.3, 0.4, 0.5, 0.6, 0.8, 1.0, and 1.2 mM CoCl_2_.

### Establishment of an OGD/R injury model in H9c2 cells

The experiment was divided into three groups: The control group, the 1%O_2_-OGD/R group, and the CoCl_2_-OGD/R group. H9c2 cells in the logarithmic growth phase were seeded into six-well plates, and the medium was discarded after 24 hr of incubation at 37 °C, 5% CO_2_. It was replaced with 1% FBS medium to synchronize cells for 24 hr. Then, the medium was replaced by a sugar-free medium containing 1% FBS medium, and meanwhile, H9c2 cells were administrated hypoxia for 16 hr at 37 °C three-gas incubator (94% N_2_, 5% CO_2_, 1% O_2_) or treatment with 0.6 and 0.8 mM CoCl_2_, and subsequently the medium was replaced by high-sugar medium containing 1% FBS to reoxygenation, and glucose for 4 hr.

### Experimental design

H9c2 cells in the logarithmic growth phase were seeded in six-well plates, and the medium was discarded after 24 hr of incubation at 37 °C, 5% CO_2_. It was replaced with 1% FBS medium to synchronize cells for 24 hr. Cell transfection: Lipofectamin^TM^2000 loaded with miR-448 mimic/inhibitor or NC was used to transfect H9c2 cells for 6 hr transiently. The medium was discarded using 2 ml 1×PBS to rinse twice. Then, 1% FBS medium was added to incubate for 24 hr. EX527/CA-5f pretreatment: The cells were pretreated with 10 μM EX527/CA-5f or dimethyl sulfoxide (DMSO) for 24 hr after cell transfection. API pretreatment: the cells were pretreated with 10 μM API or DMSO for 24 hr after EX527/CA-5f pretreatment. Subsequent treatment was performed using the OGD/R method mentioned above. The primers of miR-448 mimic/inhibitor or NC were: 

miR-448 mimic-Sence          5'-UUGCAUAUGUAGGAUGUCCCA-3'

miR-448 mimic-Antisence          5'-GGACAUCCUACAUAUGCAAUU-3'

miR-448 mimic NC-Sence          5- UUCUCCGAACGUGUCACGUTT-3

miR-448 mimic NC-Antisence.          5- UUCUCCGAACGUGUCACGUTT-3

miR-448 inhibitor-Sence          5- ACGUGACACGUUCGGAGAATT-3'

miR-448 inhibitor NC-Sence           5'-UGGGACAUCCUACAUAUGCAA-3'

The MTT assay          5'-CAGUACUUUUGUGUAGUACAA-3'

### The MTT assay

H9c2 cells in the logarithmic growth phase were seeded in 96-well plates at 8×10^3^ per well. After reagent administration, the medium was discarded, and 100 μl MTT solution was added to incubate for 4 hr. The MTT solution was discarded, and 100 μl DMSO solution was added. The plates were then shaken in a table concentrator for 10 min. Finally, the absorbance value per well was measured at a wavelength of 490 nm using a microplate reader.

### The SOD measurement assay

H9c2 cells in the logarithmic growth phase were seeded in six-well plates, and the cells were collected in a centrifuge tube after reagent administration. The centrifugation procedure was 1000 rpm for 5 min. The supernatant was discarded, and the cell sediment was suspended using 100 μl 1×PBS. Cells were disrupted by ultrasound at 300 W for 2 min, centrifugation procedure: 4 °C, 12000g, 10 min. The BCA method was performed to measure the protein concentration of the supernatant. In this assay, control wells, control blank wells, measurement wells, and measurement blank wells were set up. The reagent and sample were added according to the instructions. After completion of the reaction, the absorbance value was measured at 450 nm using the microplate reader. The SOD activity was calculated according to the SOD activity measurement formula in the instruction manual, SOD inhibitor (%) = ((A control – A control blank) – (A measure – A measure blank)) × 100%/ (A control – A control blank); SOD activity (U/mgprot) = SOD inhibitor (%) ÷ 50% × reaction system/ dilution multiple ÷ the protein concentration of the sample.

### The LDH measurement assay

H9c2 cells in the logarithmic growth phase were seeded into six-well plates. After reagent administration, the cell supernatant of the cells was collected. Blank, standard, measurement, and control wells were included in this assay. The reagent and sample were added according to the instructions. After completion of the reaction, the absorbance value was measured at 450 nm using the microplate reader. The LDH activity was calculated according to the LDH activity measurement formula in the instruction manual, LDH(U/L) = (A measurement – A control)/(A standard – A blank).

Quantitative reverse transcriptase-polymerase chain reaction (RT-qPCR) assay

H9c2 cells in the logarithmic growth phase were seeded into six-well plates. After reagent administration, the medium was discarded. Each well was rinsed twice with 2 ml 1×PBS. One milliliter Trizol reagent was added, and the cells were scraped off. The suspension was collected in enzyme-free tubes and deposited for 5 min at room temperature. 200 μl chloroform was added and shaken sharply for 15 sec, followed by incubation at room temperature for 3 min. Centrifugation procedure: 4 °C, 12000g, 15 min. The 500 μl upper water phase was carefully removed into new tubes. 500 μl isopropanol was added and shaken sharply for 15 sec, followed by incubation at room temperature for 10 min. Centrifugation procedure: 4 °C, 12000g, 10 min. The supernatant was discarded, and 1 ml of 75% ethanol was added. Centrifugation procedure: 4 °C, 7500g, 5 min. The supernatant was discarded and incubated for 10 min at room temperature. 15 μl RNase-free water was added to obtain the RNA solution sample. According to the RNA reverse transcription kit, the RNA was reverse transcribed to cDNA in the real-time qPCR instrument. The cDNA samples were amplified using the real-time qPCR instrument. The primers were:

SIRT-F           5'-UUGCAUAUGUAGGAUGUCCCA-3'

SIRTI-R           5'-TCCTTGGATTCCTGCAACC-3'

### Western blot assay

H9c2 cells in the logarithmic growth phase were seeded into six-well plates. After reagent administration, the medium was discarded. Each well was rinsed twice with 2 ml 1×PBS. 200 μl cell lysis solution (RIPA: PMSF = 100: 1) was added, and the cells were scraped off. The cells were disrupted by ultrasound at 300 W for 2 min. Centrifugation procedure: 4 °C, 12000g, 10 min. The supernatant was collected, and 5×loading buffer was added, followed by boiling at 100 °C for 10 min to obtain the protein sample. Primary antibodies: HIF-1α (rabbit, 1: 500), SIRT1(rabbit, 1: 500), p62 (rabbit, 1: 500), LC3 (rabbit, 1: 500), Bcl-2 (mouse, 1: 500), Bax (rabbit, 1: 500), β-actin (rabbit, 1: 10000). Secondary antibodies: HRP conjugated anti-rabbit IgG and anti-mouse IgG (1:2000). Data were normalized to β-actin and were disposed by ImageJ software.

### ELISA assay

Caspase-3 protein expression in the cell supernatant was measured by ELISA. H9c2 cells in the logarithmic growth phase were seeded into six-well plates. After reagent administration, the cell supernatant was collected as a sample. The standard and sample well were set up in this assay. The reagent and sample were added according to the instructions. At the end of the reaction, the absorbance value was measured at 450 nm using the microplate reader.

### Statistical analysis

All data are presented as mean ± standard deviation. One-way analysis of variance was used to compare the differences between more than two groups. The paired comparison was performed using the SNK approach. The Graph Pad Prism 9.0 software was used to process the data, and *P*<0.05 indicates a statistically significant difference.

## Results

### Molecular docking

**Figure 1:**
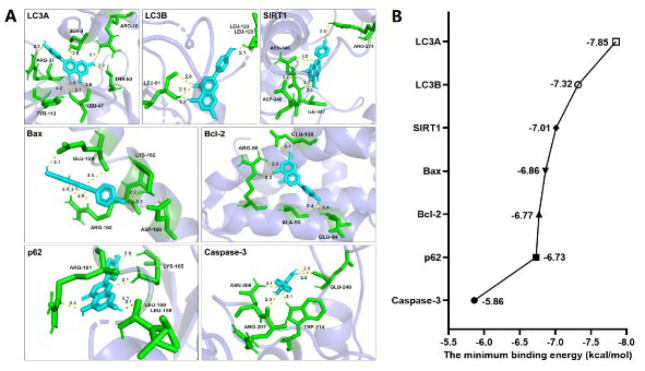
Molecular docking results for the API and target proteins

First, we used molecular docking technology to evaluate the binding affinity of API and target proteins. The docking results revealed that API with LC3A, LC3B, SIRT1, Bax, Bcl-2, p62, and caspase-3 had low binding energies, which were -7.85, -7.32, -7.01, -6.86, -6.77, -6.73, -5.86 kcal/mol, respectively (Figure 1). These data showed that the API with target proteins had strong binding ability (23).

### Establishment of an OGD/R injury model in H9c2 cells

**Figure 2:**
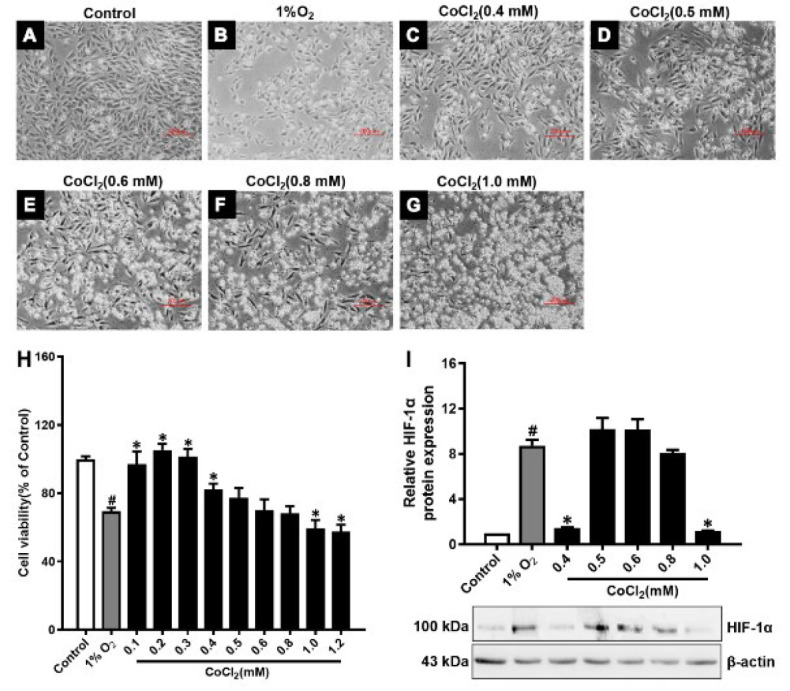
Determination of the simulated low oxygen concentration of CoCl_2_ in H9c2 cells

**Figure 3:**
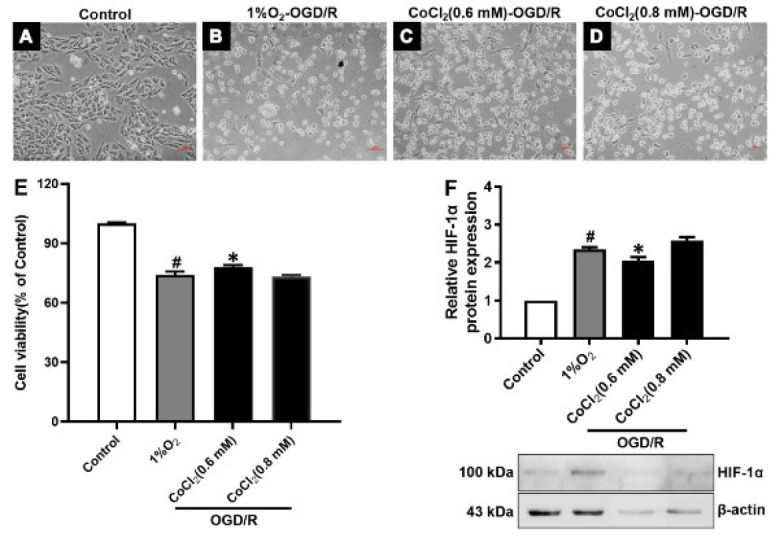
Establishment of an OGD/R injury model in H9c2 cells

To establish the OGD/R injury model of H9c2 cells, we first investigated the appropriate concentration of CoCl_2_ to simulate low oxygen. The MTT assay results showed that the cell viability of the 0.5, 0.6, and 0.8 CoCl_2 _groups was not a notable difference compared with the 1%O_2_ group (Figure 2H). The western blot results showed that HIF-1α protein expression had a consistent trend (Figure 2I). Thus, 0.6 and 0.8 CoCl_2 _were used to simulate the low oxygen conditions below. We used 0.6 and 0.8 CoCl_2_ and the sugar-free medium to construct oxygen and glucose deprivation circumstances, and then oxygen and glucose were restored. The MTT results showed that the cell viability of the 0.6 CoCl_2_-OGD/R group was not a notable difference compared with the 1%O_2_-OGD/R group (Figure 3E). The HIF-1α protein expression is the same (Figure 3F). Thus, the 0.6 CoCl_2_ was chosen to establish the OGD/R injury model in H9c2 cells.

### API promotes autophagy and inhibits apoptosis in the OGD/R model

**Figure 4:**
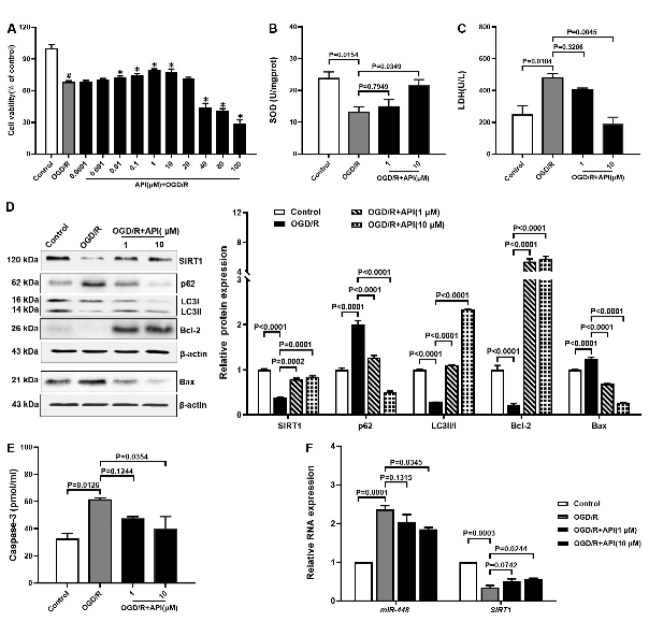
API promotes autophagy and inhibits apoptosis in the OGD/R model

To clarify the effect of API on autophagy and apoptosis in the OGD/R model, we used different concentrations of API to pretreat H9c2 cells. The MTT results showed that 1, 10 μM API can notably promote cell viability (Figure 4A). Thus, 1, 10 μM API were selected for subsequent research. Oxidative stress is one of the pathophysiological factors of MIRI. SOD is an important anti-oxidant enzyme that can scavenge superoxide and convert it to hydrogen peroxide to protect cells from oxidative stress injury. The relative study found that SOD activity was reduced in IRI (24). LDH is an enzyme that is constantly present in the cytoplasm of cells. A damaged cell membrane induces the release of LDH. The SOD assay results showed that the SOD level in the OGD/R group decreased compared with control, increased SOD level with 10 μM API, and 1 μM API failed to increase SOD level (Figure 4B) notably. The LDH data presented an opposite tendency with SOD results (Figure 4C). To investigate the effect of API on autophagy and apoptosis in the OGD/R model, we measured the expression of autophagy-related protein (SIRT1, p62, and LCII/I) and apoptosis-related protein (Bcl-2, Bax, and caspase-3). Western blot and ELISA results found decreased protein expression of SIRT1, LCII/I, and Bcl-2 but increased protein expression of p62, Bax, and caspase-3 in the OGD/R group compared with control, but the opposite results were seen in H9c2 pretreated with API, and 10 μM API has stronger effect compare with 1 μM API (Figure 4 D-E). Furthermore, we used RT-qPCR to investigate the mRNA expression of SIRT1 and miR-448, and it was found that decreased mRNA expression of SIRT1 and increased mRNA expression of miR-448 in the OGD/R group compared with control, but 10 μM API could notably rescue these results (Figure 4F). These results indicated that 10 μM API promoted autophagy and inhibited apoptosis in the OGD/R model**.**

### The miR-448/SIRT1 axis affects autophagy and apoptosis in the OGD/R model

**Figure 5:**
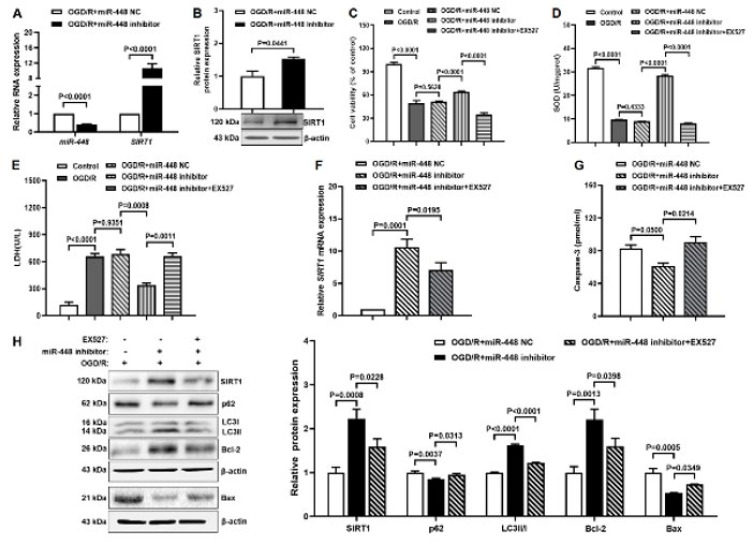
The miR-448/SIRT1 axis affects autophagy and apoptosis in the OGD/R model

To determine the relationship and function of miR-448 and SIRT1 in the OGD/R model, the miR-448 inhibitor and SIRT1 inhibitor EX-527 were administrated, respectively. SIRT1 expression notably increased in the miR-448 inhibitor group compared with the miR-448 NC group by RT-qPCR and western blot, but miR-448 notably decreased by RT-qPCR (Figure 5 A-B). Furthermore, multiple studies have demonstrated that SIRT1 was a target gene of miR-448 by Dual-luciferase reporter assay (25-27). Thus, miR-448 might negatively target SIRT1 in the OGD/R model. Increased cell viability and SOD level but decreased LDH level in the miR-448 inhibitor group compared with the miR-NC group; the opposite results were seen in H9c2 pretreated with EX-527 (Figure 5 C-E). Western blot and ELISA results found increased protein expression of SIRT1, LCII/I, and Bcl-2 and decreased protein expression of p62, Bax, and caspase-3 in the miR-448 inhibitor group compared with miR-NC group, the opposite results were seen in H9c2 pretreated with EX-527 (Figure 5 G-H). Furthermore, the RT-qPCR data of SIRT1 were consistent with the Western blot results (Figure 5F). These results indicated that the miR-448/SIRT1 axis affected autophagy and apoptosis in the OGD/R model.

### API regulates autophagy and apoptosis via miR-448 in the OGD/R model

**Figure 6:**
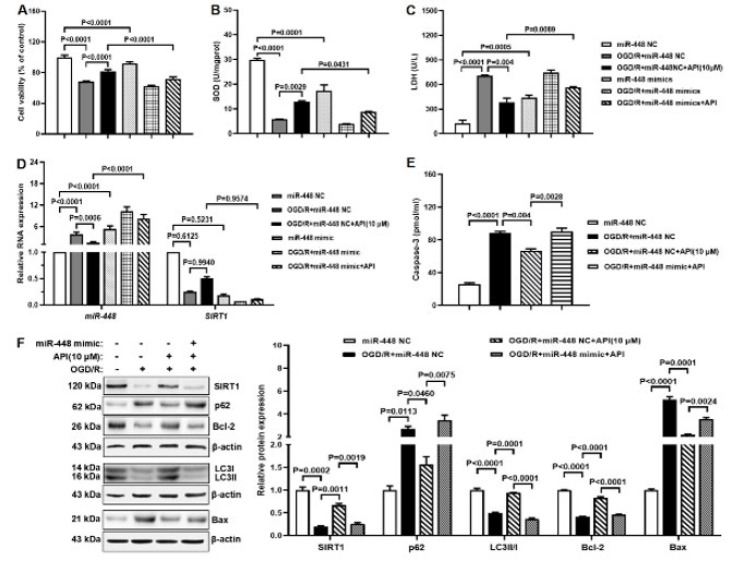
API regulates autophagy and apoptosis via miR-448 in the OGD/R model

To investigate whether API regulates autophagy and apoptosis via miR-448 in the OGD/R model, the miR-448 mimic was administrated. Compared to the OGD/R+miR-448 NC group, cell viability and SOD level elevated, but LDH level declined in the OGD/R+miR-448 NC+API group. These results were partly rescued pretreated with miR-448 mimic (Figure 6 A-C). RT-qPCR results found declined gene expression of miR-448 but elevated SIRT1 in OGD/R+miR-448 NC+API group compared with OGD/R+miR-448 NC group, but miR-448 mimic partly rescued these results (Figure 6D). Western blot and ELISA results found increased protein expression of SIRT1, LCII/I, and Bcl-2 and decreased protein expression of p62, Bax, and caspase-3 in the OGD/R+miR-448 NC+API group compared with the OGD/R+miR-448 NC group. Likewise, the miR-448 mimic partly rescued these results (Figure 6 E-F). These results indicated that API regulated autophagy and apoptosis via miR-448 in the OGD/R model.

### API regulates autophagy and apoptosis via SIRT1 in the OGD/R model

**Figure 7:**
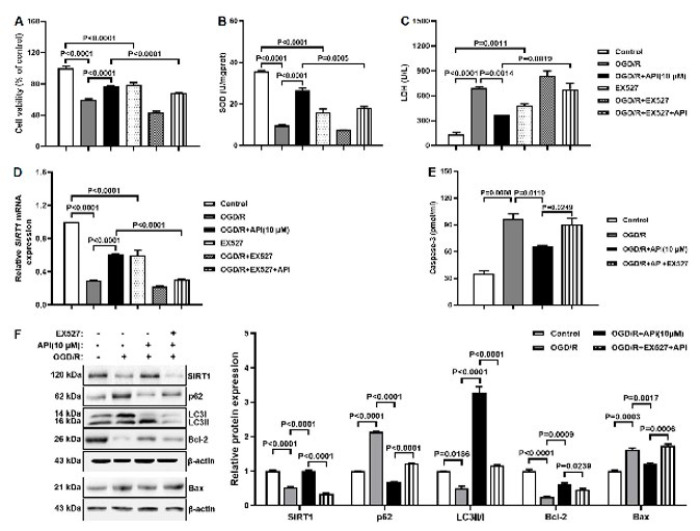
API regulates autophagy and apoptosis via SIRT1 in the OGD/R model

To investigate whether API regulates autophagy and apoptosis via SIRT1 in the OGD/R model, the SIRT1 inhibitor EX-527 was administrated. Compared to the OGD/R+API group, cell viability and SOD level declined, but the LDH level elevated in the OGD/R+EX527+API group (Figure 7 A-C). Western blot and ELISA results found declined protein expression of SIRT1, LCII/I, and Bcl-2 and elevated protein expression of p62, Bax, and caspase-3 in the OGD/R+EX527+API group compared with the OGD/R+API group, and the RT-qPCR data of SIRT1 were consistent with the Western blot results (Figure 7 D-F). These results indicated that API regulated autophagy and apoptosis via SIRT1 in the OGD/R model.

### API regulates apoptosis through autophagy in the OGD/R model

**Figure 8:**
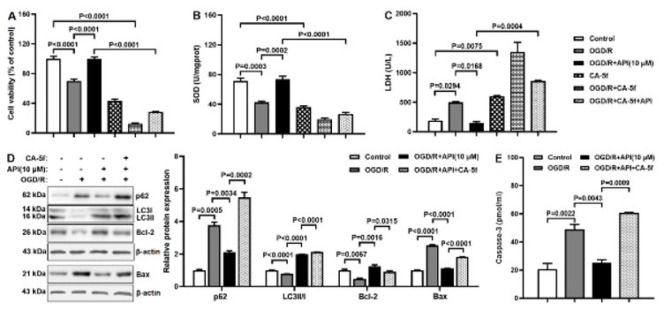
API regulates apoptosis through autophagy in the OGD/R model

To investigate whether API regulates apoptosis through autophagy in the OGD/R model, the autophagy inhibitor CA-5f was administrated. Compared to the OGD/R+API group, cell viability and SOD level declined, but the LDH level elevated in the OGD/R+CA-5f+API group (Figure 8 A-C). Western blot and ELISA results found declined protein expression of Bcl-2 and elevated protein expression of p62, LCII/I, Bax, and caspase-3 in the OGD/R+CA-5f+API group compared with the OGD/R+API group (Figure 8 D-E). These results indicated that API regulated apoptosis through autophagy in the OGD/R model. Thus, it was inferred that API regulated autophagy and apoptosis against MIRI via miR-448/SIRT1 axis.

## Discussion

Non-coding RNAs (ncRNAs) play a crucial role in developing various diseases, including miRNAs, long non-coding RNAs, and circular RNAs, and numerous studies have reported that ncRNAs are involved in the pathological development of MIRI (28). Several studies have found that miR-448 expression in IRI models shows a notable discrepancy using bioinformatics analysis, which has been demonstrated *in vitro* and *in vivo *(14, 29). Furthermore, autophagy and cardiomyocyte apoptosis are important pathological factors in MIRI (30). Thus, we designed this research to determine whether API regulates autophagy and apoptosis against MIRI via miR-448/SIRT1 axis. Our results found that API regulated autophagy and apoptosis against MIRI via miR-448/SIRT1 axis.

First, molecular docking results showed that API had a strong affinity for autophagy-related proteins (SIRT1, p62, and LCII/I) and apoptosis-related proteins (Bcl-2, Bax, and caspase-3), suggesting that these proteins were involved in the process of API against MIRI. SIRT1 is a sirtuin family member and regulates autophagy by deacetylating histone and non-histone proteins. Studies have shown that SIRT1 acetylates LC3, ATGs, and FOXOs to regulate autophagy (20). p62 is an autophagy receptor protein that inhibits autophagy by selectively binding to LC3 (31). In the autophagy process, the LC3 protein is cleaved into LC3-I, located in the cytoplasm, and subsequently, LC3-I is converted into LC3-II, located in the autophagosome membranes. It is an autophagy signal that converts LC3-I to LC3-II (32). In addition, Bcl-2 is an anti-apoptotic protein, whereas Bax and caspase-3 are pro-apoptotic proteins. The MTT assay was performed to investigate the effect of API on MIRI. The results showed that 1, 10 μM API significantly promoted cell viability in the OGD/R model. In addition, the SOD assay and the LDH assay indicated that 10 μM API significantly inhibited oxidative stress injury and cell damage. Several studies consistently reported that API can attenuate MIRI (10, 11). Liu *et al*. found that API prevented arrhythmia and myocardial pathological damage in rats and reduced apoptosis and necrotic myocardial area (33). These results indicated that API had an excellent effect against MIRI. 

To clarify the potential mechanism of API against MIRI, the miR-448 inhibitor and mimic were used. The results indicated that API regulated autophagy and apoptosis via miR-448 in the OGD/R model. Cell death has several patterns, including apoptosis, necrosis, autophagy, pyroptosis, and ferroptosis (34). There have been numerous studies of medical herbs against MIRI via regulation of cell death. Galangin is a natural flavonoid, a study found that galangin suppressed ferroptosis against MIRI via regulating the nucleus factor erythroid 2-related factor 2 (Nrf2)/glutathione peroxidase 4 (GPX4) signaling pathway (35). Hydroxysafflor yellow A is the main active ingredient of the herbal medicine safflower, which inhibits ferroptosis to attenuate MIRI by regulating the HIF-1α/solute carrier family 7 member 11 (SLC7A11)/GPX4 signaling pathway (36). Besides, a study reported that API promoted the apoptosis of colon cancer cells by inhibiting autophagy (37). Research found that API inhibited the pyroptosis of hepatocytes by promoting autophagy (38). However, no study has reported that API attenuated MIRI by regulating cell death. Our research found that API attenuated MIRI by regulating autophagy and apoptosis.

To further understand the potential mechanism of API against MIRI, the SIRT1 inhibitor EX-527 and autophagy inhibitor CA-5f were administrated. The results indicated that API regulated autophagy and apoptosis against MIRI through miR-448/SIRT1 axis. Recently, Feng *et al*. found that Qingjie Huagong decoction inhibited pancreatic acinar cell pyroptosis by regulating the circHipk3/miR-193a-5p/pyrin domain-containing protein 3 (NLRP3) pathway (39). Yan M *et al*. found that Saikosaponin D attenuated osteoarthritis by inhibiting inflammatory responses and promoting autophagy via regulation of miR-199-3p/transcription factor-4 (TCF4) axis (40). Li W *et al*. found that Curcumin inhibited prostate cancer by suppressing the proliferation and promoting apoptosis via regulation of miR-483-3p/ubiquitin-conjugating enzyme E2 C (UBE2C) axis (41). Furthermore, research found that Suxiao Jiuxin Pill alleviated MIRI by attenuating autophagy via regulating the miR-193a-3p/Alk B homolog 5 (ALKBH5) axis (42). The above studies confirmed that herbal medicine affects disease progression by regulating cell death and miRNAs.

Our study still has limitations. It only demonstrates experimental results *in vitro* using H9c2 cells, and these results should be further verified *in vivo*.

(A) Visualized figures between API and target protein in minimum binding energy. Blue is the target protein; cyan is API; green is the protein structure interacting with API; yellow is the hydrogen bond. (B) Minimum binding energy between API and target protein

(A-G) Morphology of H9c2 cells under different conditions. The experiment was divided into three groups: The control group, the 1% O_2_ group, and the different concentration CoCl_2_ group. The H9c2 cells were administrated hypoxia for 24 hr at 37 °C three-gas incubator (94% N2, 5% CO_2_, 1% O_2_) or treatment with 0.1, 0.2, 0.3, 0.4, 0.5, 0.6, 0.8, 1.0, and 1.2 mM CoCl_2_. (H) Viability of H9c2 cells was measured by MTT assay. (I) Protein expression level of HIF-1α was measured by western blot assay.

(A-D) Morphology of H9c2 cells under different conditions. The experiment was divided into three groups: The control group, the 1%O2-OGD/R group, and the CoCl_2_-OGD/R group. After the H9c2 cells were synchronized, the medium was replaced with a sugar-free medium containing 1% FBS medium. Meanwhile, the H9c2 cells were administrated hypoxia for 16 hr at 37 °C three-gas incubator (94% N2, 5% CO_2_, 1% O_2_) or treatment with 0.6 and 0.8 mM CoCl_2_, and then the medium was replaced with high-sugar medium containing 1% FBS to reoxygenation, and glucose for 4 hr. (E) The viability of H9c2 cells was measured by MTT assay. (F) Protein expression level of HIF-1α was measured by western blot assay.

(A) After H9c2 cells were pretreated with 0.0001, 0.001, 0.01, 0.1, 1, 10, 20, 40, 80, and 100 μM API or DMSO for 24 hr, the viability of H9c2 cells was measured by MTT assay. (B-C) After H9c2 cells were pretreated with 1, 10 μM API or DMSO for 24 hr, the oxidative stress injury and cell damage of H9c2 cells were measured by SOD assay and LDH assay, respectively. (D-E) Protein expression levels of autophagy-related protein (SIRT1, p62, and LCII/I) and apoptosis-related protein (Bcl-2, Bax, and Caspase-3) were measured by western blot and ELISA assays. (F) Gene expression levels of miR-448 and SIRT1 were measured by RT-qPCR assay.

(A) After H9c2 cells were transiently transfected with miR-448 inhibitor or NC for 6 hr, the gene expression levels were measured by RT-qPCR assay. (B) Protein expression level of SIRT1 was measured by western blot assay. (C-E) Cells were pretreated with 10 μM EX527 or DMSO for 24 hr after cell transfection. Cell viability, oxidative stress injury, and cell damage of H9c2 cells were measured by MTT assay, SOD assay, and LDH assay, respectively. (F) Gene expression level of SIRT1 was measured by RT-qPCR assay. (G-H) Protein expression levels of autophagy-related protein (SIRT1, p62, and LCII/I) and apoptosis-related protein (Bcl-2, Bax, and caspase-3) were measured by western blot and ELISA assays.

(A-C) H9c2 cells were transiently transfected with miR-448 mimic or NC for 6 hr. Cells were pretreated with 10 μM API or DMSO for 24 hr after cell transfection. Cell viability, oxidative stress injury, and cell damage of H9c2 cells were measured by MTT assay, SOD assay, and LDH assay, respectively. (D) Gene expression levels of miR-448 and SIRT1 were measured by RT-qPCR assay. (E-F) Protein expression levels of autophagy-related protein (SIRT1, p62, and LCII/I) and apoptosis-related protein (Bcl-2, Bax, and Caspase-3) were measured by western blot and ELISA assays.

(A-C) The H9c2 cells were pretreated with 10 μM EX527 or DMSO for 24 hr. Cells were pretreated with 10 μM API or DMSO for 24 hr after EX527 pretreatment. Cell viability, oxidative stress injury, and cell damage of H9c2 cells were measured by MTT assay, SOD assay, and LDH assay, respectively. (D) Gene expression levels of SIRT1 were measured by RT-qPCR assay. (E-F) Protein expression levels of autophagy-related protein (SIRT1, p62, and LCII/I) and apoptosis-related protein (Bcl-2, Bax, and Caspase-3) were measured by western blot and ELISA assays.

(A-C) The H9c2 cells were pretreated with 10 μM CA-5f or DMSO for 24 hr. Cells were pretreated with 10 μM API or DMSO for 24 hr after CA-5f pretreatment. Cell viability, oxidative stress injury, and cell damage of H9c2 cells were measured by MTT assay, SOD assay, and LDH assay, respectively. (D-E) Protein expression levels of autophagy-related protein (p62 and LCII/I) and apoptosis-related protein (Bcl-2, Bax, and Caspase-3) were measured by western blot and ELISA assays.

## Conclusion

Our research suggests that API regulates autophagy and apoptosis against MIRI via the miR-448/SIRT1 axis. To date, few studies have been conducted on API against MIRI, and our study discovers a novel mechanism underlying the protective effects of API against MIRI. This research lays a clinical foundation for API against MIRI. The results of this research will be further validated in animal studies in the future.

## Declaration

We have not used any AI tools or technologies to prepare this manuscript.
